# Phytochemical Fortification in Fruit and Vegetable Beverages with Green Technologies

**DOI:** 10.3390/foods10112534

**Published:** 2021-10-21

**Authors:** Francisco Artés-Hernández, Noelia Castillejo, Lorena Martínez-Zamora, Ginés Benito Martínez-Hernández

**Affiliations:** Department of Agronomical Engineering & Institute of Plant Biotechnology, Universidad Politécnica de Cartagena, 30203 Cartagena, Spain; noelia.castillejo@upct.es (N.C.); lorena.martinez@upct.es (L.M.-Z.); ginesbenito.martinez@upct.es (G.B.M.-H.)

**Keywords:** smoothies, juices, elicitors, abiotic stresses, nutraceuticals, health-promoting compounds

## Abstract

Background: Phytochemical, bioactive and nutraceutical compounds are terms usually found in the scientific literature related to natural compounds found in plants linked to health-promoting properties. Fruit and vegetable beverages (mainly juice and smoothies) are a convenient strategy to enhance the consumption of horticultural commodities, with the possibility of being fortified with plant byproducts to enhance the content of bioactive compounds. Objective: This review aims to analyse the different green technologies applied in beverage processing with a fortification effect on their health promoting compounds. Results: Fortification can be performed by several strategies, including physical elicitors (e.g., processing technologies), plant/algae extract supplementation, and fermentation with probiotics, among others. Thermal processing technologies are conventionally used to ensure the preservation of food safety with a long shelf life, but this frequently reduces nutritional and sensory quality. However, green non-thermal technologies (e.g., UV, high-pressure processing, pulsed electric fields, ultrasounds, cold plasma, etc.) are being widely investigated in order to reduce costs and make possible more sustainable production processes without affecting the nutritional and sensory quality of beverages. Conclusions: Such green processing technologies may enhance the content of phytochemical compounds through improvement of their extraction/bioaccessibility and/or different biosynthetic reactions that occurred during processing.

## 1. Introduction

More than two millennia ago, the father of modern medicine related the use of appropriate foods for therapeutic benefits (Hippocrates, 460–377 BC). In this sense, the origin of the term ‘nutraceutical’, first coined by Stephen DeFelice in 1989, comes from the combination of nutrients and pharmaceuticals, because these compounds have been shown to provide medical or health benefits, including the prevention and the treatment of disease [1]. Nevertheless, a regulatory definition has not been reached yet. As synonyms of this terminology, EFSA has defined a ‘bioactive compound’ as a type of chemical found in small amounts in plants and certain foods (such as fruits, vegetables, nuts, oils, and whole grains) and which have actions in the body that may promote good health [2]. In this sense, the term ‘phytochemical’ also refers to nutritive or non-nutritive, biologically active compounds present in edible natural foods, including fruit, vegetables, grains, nuts, seeds, and tea, which also prevent or delay chronic diseases in humans and animals [3]. Nevertheless, an EFSA or USDA positive opinion regarding a bioactive compound implies the need to corroborate a clear relationship based on clinical studies in healthy consumers. Therefore, the use and development of these substances and their incorporation into foods and beverages depends on their safety, adverse effects, and toxicity studies [4].

Healthy habits such as diet and active lifestyle are associated with human wellbeing. Nowadays, people have become concerned about their health and dietary habits, especially since the COVID-19 pandemic. Nutraceutical compounds contained in foods have gained increasing attention because they can provide health benefits with negligible side effects compared to traditional pharmacological therapies. Therefore, although there is no evidence on their effects as a potentially useful against SARS-CoV-2 infection, consumers are focusing on proper nutrition rich in antioxidant nutrients [5]. In this context, fruit and vegetable beverages are excellent and convenient drinks to promote the consumption of bioactive compounds. Common types of fruit and vegetable beverages mostly include juices and smoothies. While a juice usually contains the liquid extracted when fruit and vegetables are pressed and the pulp is discarded, smoothies are made of whole products in a blender, usually containing some non-juiceable ingredients. Rodríguez-Verástegui et al. [6] have defined smoothies as non-alcoholic beverages prepared from fresh or frozen fruits and or vegetables, which are blended and usually mixed with crushed ice to be immediately consumed. The main difference is fibre, which makes you feel full, which smoothies contain much of while juices typically have very small amounts. While by-products are discarded in juices, smoothies usually contain the whole edible product, which includes important bioactive compounds. Hence, high health benefits can be obtained from mixing fruit and vegetables due to the synergistic relationships between their different bioactive compounds [7]. As the conversion of fruit and vegetables into processed products also enhances the economic value of fruit, the fruit and vegetable beverages industry is rapidly growing worldwide [8].

The main issue in smoothie/juice processing is their limited shelf-life; they are susceptible to spoilage [9] and quality degradation. Moreover, quality degradation due to endogenous enzyme activity is an important factor, as while in preparation enzymes can come in contact with substrates. For this reason, thermal treatments are usually performed during processing in order to increase shelf life while keeping quality and avoiding microbial spoilage [10]. However, the application of heat treatments for long times negatively affects the nutritional and sensory quality of smoothies and juices [11]. Given the high economic and environmental impact of such technologies, alternative technologies have been recently developed to reduce costs while maintaining a high content of health-promoting compounds. In this field, new emerging eco-friendly technologies are expected to be developed during the coming years.

The horticultural industry generates a large number of by-products that constitute an excellent source of valuable bioactive compounds which deserve to be revalorized. Therefore, green extraction techniques have been developed in recent years and innovative additional systems for phytochemical extraction in fruit and vegetable beverages have been performed [12].

Therefore, this work reviews the nutraceutical fortification of fruit and vegetable beverages by using green emerging technologies to improve the bioactive content and preserve sensory quality and microbial safety while reducing costs and energy resources.

## 2. Nutraceutical Food Supplements

Nutraceuticals can be classified based on their natural source (from plants, animals, or microbes), their mechanism of action (antioxidant, anti-inflammatory, and anticancer capacities, mainly), or their chemical constitution [4]. With respect to their main components in terms of chemical structure, the most relevant nutraceutical compounds are carotenoids, dietary fibre, omega-3 fatty acids, flavonoids, glucosinolates, indoles, isothiocyanates, phenolic acids, plant sterols, prebiotics, probiotics, saponins, phytoestrogens, tannins, thiols, and sulphides [4]. In this context, many of these bioactive compounds can protect our bodies against disease as well as prevent food spoilage by blocking oxidation processes. These reactions are performed in different ways: preventing chain inhibition by scavenging initiating radicals, acting as electron donors, breaking chain reactions, transferring hydrogen atoms to generate stable radicals, decomposing peroxides, decreasing localized oxygen concentrations, and binding chain initiating catalyst such as metal ions [13].

The adherence to the Mediterranean dietary pattern, characterized by the presence of foods rich in such bioactive compounds, has been demonstrated to have potentially beneficial health effects and can have cardioprotective, neuroprotective, antioxidant, anti-inflammatory, and anticarcinogenic properties [14].

**Figure 1:**
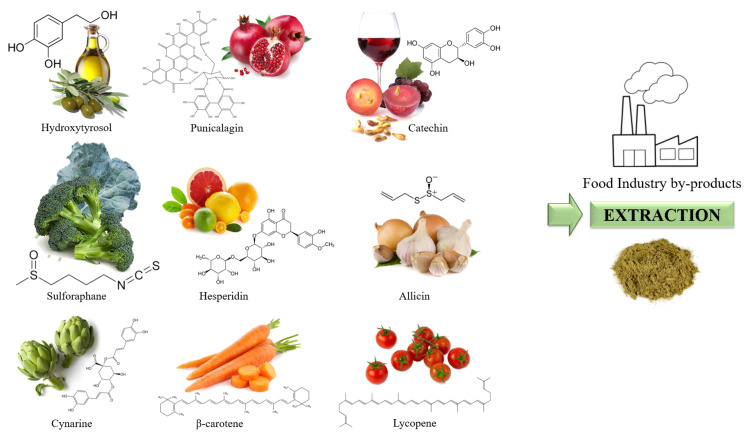
Some of the most common nutraceutical compounds obtained as food industry by-products used to enrich fruit and vegetable beverages.

Food industry by-products produced in the Mediterranean basin, as an important production area of fruit and vegetables consumed worldwide, have become an important source of nutraceutical supplements used as enrichers and fortifiers in food and beverages (Figure 1). As examples, olive oil production is the main source of natural hydroxytyrosol (Figure 1), one of the most important antioxidants in the “Mediterranean Diet”, even more than tea or Q10 coenzyme [15]. Similarly, the production of wine is an inexhaustible source of flavonoids, catechins and epicatechins obtained from grape seed and grape byproducts (Figure 1). Additionally, the increased production of fresh-cut and ready-to-eat convenience products obtained from fruit and vegetables, where waste accounts for more than 50%, takes a further step towards the revalorization of such amount through the extraction of their antioxidant compounds and their subsequent incorporation into food matrices. Figure 1 presents some examples of these compounds: punicalagin from pomegranate, hesperidin or naringenin from citrus peels, sulforaphane from broccoli leaves and florets, allicin from garlic and onion peels, lycopene from fried tomato or ketchup processed, β-carotene from fresh-cut carrots, and cynarine from artichokes leaves.

Furthermore, it is important to emphasise that the bioavailability of these bioactive compounds is highly elevated, which also justifies their incorporation in nutraceuticals as a way to enrich processed food and beverages. In this sense, isothiocyanates have been shown to have 37–50% bioavailability [16,17], with a demonstrated anticancer potential. In addition, carotenoids have been reported to be highly available for the human body (80–90%) [18,19], while phenolics and flavonoids have shown between 50% and 80% availability of the total consumed content [16]. Indeed, hydroxytyrosol obtained from olive leaves has shown 98.5% bioavailability in vitro [20,21].

In addition, some extraction procedures improve the uptake rate of some nutraceutical molecules, as occurs with lycopene [22,23] and some vitamins [24]. Thus, as the literature is extensive, it is necessary to review how green technologies can enhance nutraceutical content and/or availability, even after their incorporation into foods and beverages.

## 3. Preservation of Fruit and Vegetable Beverages Using Green Technologies

Despite the high nutritional value of fruit and vegetable beverages, their shelf life is limited due to processing steps in which the blending process breaks most of the cells and leaves them unprotected against microbial and enzymatic spoilage, which contribute to impairment of sensory, nutritional, and safety parameters [25]. The main strategy to preserve quality and safety is refrigeration; however, even under low storage temperatures the deterioration rate is very high.

In this sense, heat treatments such as pasteurization and sterilization have been widely used to ensure safety and prevent spoilage [26]. The main aim of these procedures is to destroy pathogenic microorganisms (*Escherichia coli*, *Staphylococcus*, *Listeria*, *Salmonella*, *Bacillus*, and *Clostridium,* among other species) and decrease spoilage organisms that grow during their shelf life. Moreover, these thermal treatments are used to inactivate enzymes such as pectin methylesterase, polyphenoloxidase, and peroxidase, which are responsible for detrimental effects on product quality.

**Figure 2:**
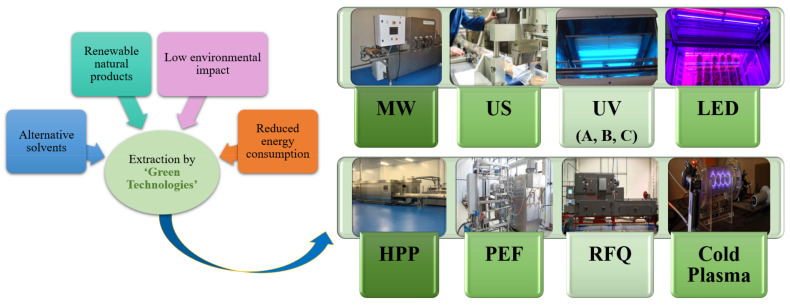
Green technologies to improve the healthiness of fruit and vegetable beverages. MW: microwave; US: ultrasounds; UV: ultraviolet; LED: Light Emitting Diode; HPP: High-Pressure Processing; PEF: Pulsed Electric Field; RFQ: Radiofrequency.

However, the application of high temperatures over long periods usually affects the nutritional and sensory quality of smoothies and juices [11]. In addition, the continuous use of thermal treatment in the Food Industry has a high economic cost and a high impact on carbon footprint. In the last few decades, alternative treatments, referred to as ‘Green Technologies’, have been developed to reduce costs and make possible more sustainable production processes. A summary of these emerging technologies, which support energy efficiency, recycling, health and safety concerns, and renewable resources, is shown in Figure 2.

### 3.1. Microwaves

Microwave (MW) processing is a thermal treatment used for the pasteurization of liquid products that can reach high temperatures in seconds, which considerably reduces application time without altering sensory and nutritional quality [11]. For instance, continuous MW heating, combining high power and short time (3.6 kW for 93 s) reaching 90 °C, inactivated up to 96% of peroxidase activity compared to conventional heating of untreated smoothies. Furthermore, this treatment increased the viscosity of a tomato smoothie [27]. In this sense, Markowski et al. [28] related this increase in viscosity as an indicator of severe heat treatment, and the highest degradation of ascorbic acid.

### 3.2. Ultrasounds

Ultrasound (US) is an emerging technology characterized by its high efficiency, low and economical instrumental requirements; it has been used in juices to increase their shelf life while maintaining their nutritional and physicochemical attributes [29]. However, it decreases the viscosity of juice due to cavitation phenomena. The Ultrasound acts on cellular structures, breaking them due to extreme conditions, mainly pressure. From an industrial point of view, a less viscous liquid should behave better under this process. For instance, 147 W US treatment for 2 min affected the viscosity of a fruit smoothie and even achieved the maximum retention of anthocyanin (99%) [30]. In addition, other authors [31] have reported that ultrasound treatment was able to retain ascorbic acid (84–91%) content even more than using pulsed electric fields (PEF) (80–83%).

### 3.3. Ultraviolet

Ultraviolet (UV) light, especially UV-C (280–100 nm), has been widely reported on for its germicidal effects against a wide range of pathogenic and food spoilage microorganisms, although recent findings have shown that low doses of UV-C are also able to enhance the nutraceutical content in acerola fruits [32]. Furthermore, UV-B (320–280 nm) and UV-A (400–320 nm) have demonstrated properties that improve the content of the bioactive compounds in broccoli sprouts [33,34], kale sprouts [35], bell peppers [36], carrots [37], and broccoli [12], among others. UV light is an environmentally friendly technology with low costs in terms of equipment, energy and maintenance. However, due to the low penetration power and transmission of UV light, the application of this technology in beverages is limited [8].

### 3.4. Light Emitting Diodes (LEDs)

A Light Emitting Diode (LED) emits different light colours depending on the energy of the photons. The technological advancements in LED technology have resulted in widespread application in horticultural production systems due to its limited thermal dissipation, low energy requirements and the possibility of finely customizing the light intensity and spectral properties [38]. For instance, past research has shown that different regions of the visible spectrum are able to affect the biosynthesis of nutraceuticals, since fruit, plants, and vegetables have photoreceptors in these wavelengths that trigger the accumulation of glucosinolates, carotenoids, phenolic acids, and flavonoids, among others.

### 3.5. High Hydrostatic Pressure Treatment

High pressure processing (HPP) is based on Le Chatelier’s principle. Low molecular weight compounds such as vitamins, minerals, and volatile compounds are rarely affected by HPP due to the low compressibility of covalent bonds. In contrast, macromolecules like proteins, lipids, or starches can similarly change their native structure under heat treatments [39]. The application of HPP in beverages ranges from 50 to 1000 MPa for several minutes and cycles, in combination with controlled temperature (<0 °C up to 100 °C). In such conditions, HPP can produce important antimicrobial effects with no consequences in the physicochemical and nutritional profile [39,40]. Furthermore, in combination with high temperatures (60–90 °C), HPP technology (300–700 MPa) can be used for food sterilisation, although this can lead to colour losses [40].

### 3.6. Pulsed Electric Fields

Pulsed electric field (PEF) treatment involves the application of direct current voltage pulses for very short periods (µs-ms), which results in an electric field the intensity of which depends on the gap between the electrodes. The microbial inactivation fundament is based on the electroporation of bacterial membranes producing pores in cellular tissues, leading to leakage of intracellular contents. As bacterial spores are resistant to PEF treatments, its application should be focused on pasteurization [41].

### 3.7. Radiofrequency

Radiofrequency (RFQ) refers to electromagnetic waves in the range of 10–300 MHz, however, the range of permitted frequencies in industrial applications is 10–50 MHz [42]. When foods with several factors affecting the electric field (food composition, salt, fat content, moisture, temperature, etc.) are exposed to an alternating electric field, dielectric heating occurs. Such heating is directly related to microbial reduction at a relatively low temperatures (60–65 °C), even able to inactivate pathogenic microorganisms such as *Listeria monocytogenes* and *E. coli* [43]. In this sense, the controlled application of this technology provides fast heating with the purpose of sterilization, pasteurization, thawing, and drying. However, lengthy exposure to high temperatures can produce loss of nutraceuticals, colour, and aroma, altering the quality of fruit and vegetable beverages.

### 3.8. Cold Plasma Treatment

Cold plasma (CP) technology provides a wide range of antimicrobial action while requiring low temperature changes and short times for application, which make it preferable for use with the thermolabile compounds responsible for the nutritional and sensory quality in fruit and vegetable beverages [44]. Plasma is the fourth state of matter, an ionized gas composed of ions, radicals (reactive oxygen and nitrogen species, ROS and RNS, respectively), atoms, and electrons in both excited and ground states [45]. These excited atoms and molecules emit excess energy in broad-spectrum electromagnetic radiation, including UV, when they return to a more stable state. The main parameters for plasma generation are pressure, voltage, treatment time, and type of gas [46]. Its application in juices is based on its ability to inactivate microorganisms located on food surfaces, food packaging materials, and process equipment under atmospheric pressure conditions [46,47].

## 4. Phytochemical Elicitors in Fruit and Vegetables Used in Beverages

Although the term ‘elicitor’ was originally applied to molecules able to induce the production of phytoalexins [48], nowadays it is used for compounds that stimulate any type of plant defence. Therefore, plant elicitors could be defined as external agents (biotic or abiotic) which act as indirect sources of enrichment and fortification due to plant response by synthetization of secondary metabolites because of the stress-induced stimulation.

In this sense, the first plant elicitors were described by Keen [49] as biotic stresses produced by pathogens. Nowadays, the exact molecules (polysaccharides, oligosaccharides, proteins, glycoproteins, or fatty acids) are independently added to produce such elicitation. However, the application of controlled postharvest abiotic stresses was proposed in 2003 by Cisneros-Zevallos [50] as an innovative tool to increase the biosynthesis of health-promoting compounds. In this review, we will focus on some of the abiotic stresses applied in raw plant materials used for beverages.

**Figure 3:**
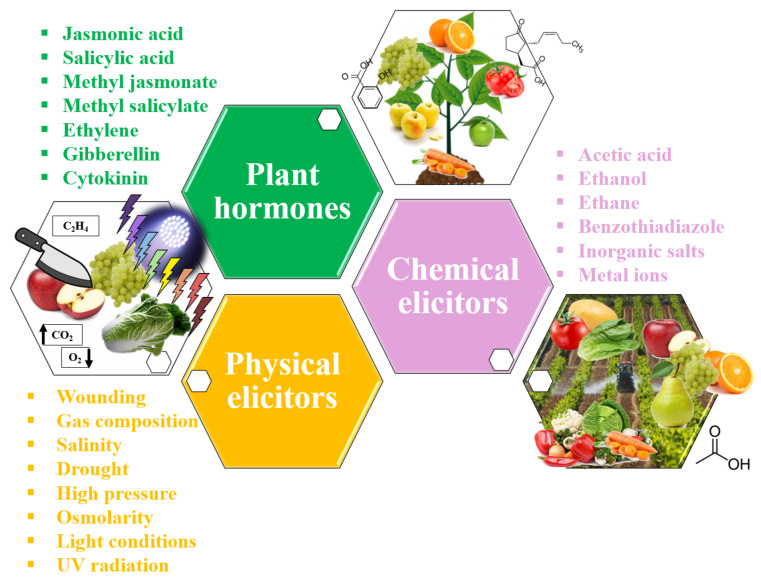
Summary of abiotic stresses applied as plant elicitors.

In this sense, abiotic stress can be classified according to its nature into three main groups: physical elicitors, chemical elicitors, and plant hormones (Figure 3).

Physical elicitation, such as wounding, temperature, gas composition, salinity, drought, high pressure, osmolarity, light conditions, and UV radiation, refers to controlled physical damage applied to the plant to trigger the activation of the plant’s secondary metabolism as a defence mechanism. To confirm this behaviour, many authors have studied the correlation between these abiotic stresses and the accumulation of nutraceuticals. For instance, Jacobo-Velázquez and Cisneros-Zevallos have shown a direct relation between wounding carrot tissues and the accumulation of chlorogenic acid and its derivatives, which highly increased in combination with high temperatures (>15 °C), UV-C light, and the application of plant hormones [51,52]. Low doses of UV-A, UV-B, and UV-C in combination with wounding in fresh-cut carrot was also effective [53,54]. In addition, these authors have shown this behaviour in red prickly pears after a combination of wounding and UV-B light [55]. Furthermore, our own findings have demonstrated that the combination of UV-B and UV-C radiation separately applied can enhance glucosinolate biosynthesis in broccoli by-products [12] and phenolics in carrots [37]; furthermore, the simultaneous application of UV-B and UV-C at low doses can increase sulforaphane in broccoli and radish sprouts [56]. Moreover, hyperoxic conditions have shown to be interesting tools for improving phenolic biosynthesis in carrots [57] and tatsoi baby leaves [58], alone or combined with UV-C radiation.

In a similar trend, acetic acid, ethanol, ethane, benzothiadiazole, inorganic salts, and metal ions have been applied to crops as chemical elicitors of major bioactive compounds to improve the quality of plants and fruits. For instance, some heavy metal salts such as AgNO_3_ and CdCl_2_ are able to trigger the production of phytoalexin and alkaloids; synthetic chemicals can also produce these signals [48].

Lastly, plant hormones such as jasmonic acid, salicylic acid, methyl jasmonate, methyl salicylate, ethylene, gibberellin, and cytokinin, among others, have also been studied as abiotic elicitors in plant cultivars. Thus, jasmonic acid, salicylic acid, and methyl jasmonate have been studied in combination with physical elicitors to increase the accumulation of glucosinolates in broccoli sprouts [34,59].

In this sense, most of the “Green technologies” previously detailed could act as inductors of the elicitation of phytochemicals in fruit and vegetables, which can be an enriched source of functional juices and beverages. In fact, these new technologies (HPP, US, UVC, PEF, RFQ, and CP) can extend the shelf-life of such products without the need to apply high temperatures, which helps to preserve their sensory and nutritional quality with no reduction in the phytochemical content.

## 5. Green Technologies as Elicitors of the Fortification of Fruit and Vegetable Beverages

### 5.1. Ultraviolet

The use of UV light to reduce microbial loads may not compromise the phytochemical content of beverages (Table 1). It is crucial to remember that several UV treatment parameters (UV dose, intensity, distance to the product, etc.) must be considered when evaluating the effectiveness of such treatments [60]. UV-C treatment at 1.08 kJ m^−2^ of kale juice, which reached up to 5-log reduction of inoculated *E. coli*, only led to low (<20%) total phenolic content (TPC) reduction [61]. High microbial inactivation (2.5–5.9 log reductions) was also found after UV-C treatment (10.6 kJ m^−2^) in inoculated *E. coli*, *Pseudomonas fluorescens* and *Saccharomyces cerevisiae* in carrot-orange juice [62], while a similar UV-C dose (11.4 kJ m^−2^) aimed to preserve the sensory quality of carrot juice during storage at 5 °C.

Higher UV-C doses in melon juice (16 kJ m^−2^) and pineapple–mango juice (8 kJ m^−2^) did not induce either TPC or total antioxidant capacity (TAC) changes [63,64]. UV-A treatment (1.5 J m^−2^) did not either affect TPC and flavonoid content, while 1 log reduction of microbial spoilage was achieved [65]. Furthermore, UV treatments better preserved TPC, TAC and physicochemical quality (colour, pH, SSC, viscosity, etc.) [61,63,64,65,66]. Interestingly, a combined UV-C/UV-A treatment led to TPC and TAC enhancements of 1.8- and 4.6-fold in carrot-carob-ginger-lemon-grape juice [66]. The better preservation of phytochemical contents in these beverages may be owed to: (i) increased phytochemical extraction; (ii) impairment of some phenolic molecules; (iii) breakdown of polyphenols into smaller phenolic components; and/or (iv) antioxidant biosynthesis as a response to the free radicals produced during UV light exposure [63,66]. On the other hand, the observed mild phytochemical reductions in some studies may be explained by oxidation reactions and double bond disruption of these compounds promoted by free radicals produced during UV treatment and further photon absorption by double bonds or oxygen [61,63].

### 5.2. High-Pressure Processing

HPP is a well-known green technology for its minimal impact on the phytochemical content of beverages. In addition, phytochemical increases may be expected due to plant cell disruption leading to higher extractability of these compounds. Nevertheless, special attention must be paid since within these disrupted cells degradative enzymes come in contact with their substrates, which is crucial to high enzyme inactivation rates. Thus, the polyphenol oxidase (PPO) activity of a fruit smoothie was inactivated by 83% after a 600 MPa-HPP treatment (5 min; 20 °C) [71]. Thus, it is recommended that HPP treatments be conducted at higher temperatures (usually up to 60 °C) to increase the inactivation rate of degradative enzymes like PPO, ascorbate oxidase, etc. In addition, unavoidable heat transfer among vessel and products through pressurisation and depressurisation, and the increased time to reach final pressurisation and additional dwell time, may also affect the phytochemical content and enzyme activity [71].

Vitamin C (ascorbic acid) is considered among the most thermolabile antioxidants in fruits and vegetables, being considered an indicator of the nutritional quality of beverages after processing treatments. HPP is considered an excellent green technology that does not greatly affect the vitamin C content of fruit and vegetable beverages, as heat treatments do. Hence, mild HPP treatments (350–450 MPa; 5 min; 10–20 °C) were enough to reach high vitamin C retention in different fruit–vegetables smoothies [68,70,71,79]. Furthermore, the dissolved oxygen proportion in beverages is of high interest due to the high oxidation rates of vitamin C. Thus, dissolved oxygen in a fruit smoothie was reduced approximately three-fold after HPP treatment (450–600 MPa; 5–10 min; 20 °C) with consequent better vitamin C retention after treatment [71].

With respect to phenolic compounds, HPP treatments (350–600 MPa; 5 min; 10–25 °C) also retained the TPC of fruit smoothies with unchanged values [69,70,77]. Furthermore, the TPC of fruit juice treated with HPP (600 MPa; 5 min; 25 °C) was only slightly reduced (≈10%), correlated with its TAC, after 30 days at 4 °C [77]. Such good phenolic retention during storage may be explained by the high PPO inactivation rates that are achieved, as previously observed [71]. Interestingly, more intense HPP treatments (500 MPa; 50 °C; 20 min) induced a TPC enhancement of 20% in gooseberry juice [76]. Such increases can be explained by several aspects: (i) enhanced solubility of phenolic compounds due to pressurization, resulting in increased antioxidant activity; and (ii) structural changes due to HPP leading to modifications in the product constituents (proteins, lipids and carbohydrates), which may allow greater access of the enzymes and promote the release of antioxidant compounds such as bioactive peptides, fat-soluble vitamins, carotenoids and polyamines [76,77]. Furthermore, phenyl ammonia lyase activity, the key enzyme in the biosynthesis pathway of polyphenols, was six-fold higher in an HPP-treated (300 MPa; 5 min; 23 °C) orange smoothie after seven days at 4 °C compared with an untreated smoothie [74]. Kouniaki et al. [113] hypothesized that the high ascorbic acid content common in fruits and vegetables may negatively impact anthocyanin content. Nevertheless, HPP treatment (600 MPa; 5 min; 25 °C) of juçara–mango juice aimed to preserve the anthocyanin content with unchanged values [77].

Carotenoid contents (α- and β-carotene) of carrot juice were better preserved with HPP (550 MPa; 6 min; <38 °C), with values 1.4–1.7-fold higher compared with heat-treated juice (110 °C; 8.6 s), which was also observed during subsequent conservation at 4 °C up to 20 days [73]. HPP treatment of whole peeled oranges (200 MPa; 1 min; 25 °C) followed by HPP of the juice (400 MPa; 1 min; 40 °C) induced 12-fold higher contents of phytoene and phytofluene, which was explained by the authors due as to the highly hydrophobic nature of these carotenoids, leading to a higher extractability after HPP and enhanced release from the cellular structures to the juice [78].

HPP has been also proposed as a green technology to reduce aflatoxins [75]. Hence, HPP treatment (500 MPa; 5 min; 45 °C) reduced aflatoxins B1/B2, G1 and G2 by 14–17%, 19%, and 29%, respectively, in grape juice [75]. The authors also stated that the effectiveness of HPP in reducing the aflatoxin content in fruit beverages may depend on the studied mycotoxin, the food matrix, and the applied conditions.

### 5.3. Pulsed Electric Fields

Treatment of fruit juice by PEF did not affect anthocyanin content [31]. Nevertheless, it is known that electroporation due to PEF treatments may enhance the extractability of bioactive compounds such as anthocyanins, although PEF may also promote reactions that reduce the content of these phytochemicals. Accordingly, when the previous authors supplemented fruit juice with an antioxidant (stevia) the anthocyanin content was hypothesized to be protected, and higher contents were observed [31]. A similar beneficial effect of stevia to preserve the anthocyanin content of fruit juice after HPP was observed by the same authors [114]. In addition, they found that the electric field, more so than treatment time, was the factor that most influenced anthocyanin content.

Both electric field and treatment time influenced the carotenoid content of fruit juice after PEF, showing lower electric fields can lead to enhancement of carotenoids, explained by higher extractability after PEF with low generation of ROS (which may promote the oxidation of the carotenoid chain) [31].

Nevertheless, PEF (20–40 kV/cm; 100–360 μs; <50 °C) of fruit juice led to ascorbic acid reductions, with higher electric fields strengths inducing larger decreases in this bioactive compound independently of the treatment time [31]. The authors explained such negative effects as being due to the higher extractability of intracellular contents after PEF due to electroporation, and the consequent higher oxidation reactions of ascorbic acid.

The aflatoxin content of beverages can also be reduced by PEF treatment, as previously reported [75]. These authors achieved 24–25%, 72% and 84% reductions of AFB1/AFG2, AFB2 and AFG1 reductions, respectively, after PEF treatment (3 kV/cm, 238 pulses; <75 °C) in grape juice. PEF induces the permeation of the cell membrane, forming membrane pores (temporarily or permanently), which may modify the structure of amino acids, proteins, and polysaccharides. Meanwhile, HPP treatment has minimal effect on the breakage of covalent bonds and is transmitted instantaneously; thus, no gradients are formed, which may explain the lower aflatoxin reduction after HPP compared with PEF found by Pallarés et al. [75].

As observed, PEF is an excellent green technology that has little effect on the content of phytochemicals in beverages while achieving good microbial reduction, extending the product shelf life. Hence, >5 log reductions of *E. coli*, *L. monocytogenes*, *Staphylococcus aureus* and *Salmonella typhimurium* were achieved in apple juice after PEF treatment (25 kV/cm; 63 μs; <55 °C) in [80], with the potential to extend the product shelf life as observed in PEF-treated fruit smoothies and juices [81,83].

### 5.4. Ultrasound

Ultrasonic processing of beverages generally enhances the content of most phytochemical compounds, such as phenolic compounds, flavonoids, carotenoids, etc., as compiled in Table 1. Thus, sonication is a well-known procedure in laboratories to improve the extraction of compounds from the food matrix.

Phenolic contents can be enhanced with US processing, as observed in fruit juices, whose TPC was increased up to 60% after treatments of 30–60 min (20–25 kHz) [90,91]. Nevertheless, shorter (<30 min) processing of beverages did not improve the TPC [88,91,92]. Such phenolic enhancements were highly correlated with TAC increases due to the highly antioxidant nature of these compounds [90,91]. Focusing on individual phenolic compounds, chlorogenic acid, caffeic acid and catechin were enhanced by 40%, 20% and 16%, while phlorizin and epicatechin increases reached up to 76% and 130% after US processing (60 min; 25 kHz) of the fruit juice [87]. Interestingly, contents of individual phenolic compounds in a nopal beverage were also enhanced after treatment, although without difference between treatment times from 10 to 40 min [91]. The observed phenolic enhancements of the authors with short treatments (10 and 20 min) similar to 40 min processing may be explained by the high frequency (42 kHz) used by those authors [91]. Similarly, treatment at 37 kHz for 10 min induced sinapic and gallic acid increases in a vegetable–coconut beverage [92]. Thus, each phenolic compound has a different sensitivity to ultrasonic waves, and its content may be increased or preserved [91]. Enhancement of flavonoids seems to be higher, since processing of just 15–30 min (20–24 kHz) induced increases of the total flavonoid content of 30–90% in several fruit juices [85,88,90].

The enhancement of phenolic compounds in beverages after US may be explained by several reasons: (i) increased extractability owing to the disruption of cell walls during the sudden change in pressure of liquid by the shear force exerted by cavitation, which can facilitate the liberation of bound polyphenolic contents; and (ii) attachment of hydroxyl radicals to the aromatic ring of phenolic compounds during sonochemical reactions occurring during US processing [84,89]. Furthermore, the addition of a second hydroxyl group to the ortho- or para-positions has previously been reported to enhance the antioxidant capacity of phenolic compounds [84]. At the same time, unchanged phenolic contents such as those observed with short US processing may be the result of their use as an antioxidant against the generation of free radicals (e.g., hydroxyl and hydrogen-free radicals) during sonication due to the dissociation of the water molecules in aqueous solutions as a result of the high temperature and pressure of the collapsing gas bubbles associated with cavitation [90].

Carotenoid contents of fruit juices were also increased after US processing [84,87,89]. In particular, the total carotenoid content of apple juice was increased by 27% after processing for 60 min (25 kHz) [84], with such enhancement of total carotenoid content of grapefruit juice increased up to 40% with a longer processing time of 90 min (28 kHz) [89]. Blanching (water bath at 100 °C for 4 min) carrots before US processing of the carrot juice improved carotenoid retention, with higher carotenoid content (1.9- and 1.7-fold higher lycopene and lutein, respectively) in the sonicated juice from blanched carrots compared to the juice from unblanched carrots [87]. These higher values after US processing when the raw product is previously blanched may be owing to the inactivation of degrading enzymes and/or additional disruption of plant cells, which enhances their extractability after the subsequent ultrasound treatment.

On the contrary, the ascorbic acid content of beverages is generally reduced under common US processing [91,92]. Thus, a 6–7% reduction in the ascorbic acid content of a vegetable–coconut beverage was observed after 10–15 min (37 kHz) of processing [92]. This ascorbic acid reduction during US processing is increased when the treatment time is augmented, as was observed in a nopal beverage after 20 and 40 min of processing (42 kHz) [91]. Interestingly, lower US frequency (24 kHz) and higher temperature (43–46 °C) during treatment, regardless of the treatment time, led to increases of the ascorbic acid content of ≈30% [90]. The latter finding may be explained by the elimination of dissolved oxygen (which leads to oxidation of ascorbic acid) during sonication being increased at higher temperatures. However, the observed ascorbic acid degradation in beverages under temperatures <30 °C may be explained by the generation of hydrogen ions (H^+^), free radicals (O^−^, OH^−^) and hydrogen peroxide (H_2_O_2_) during the sonication of water molecules, which may degrade ascorbic acid [92]. In addition, such US treatments may not be enough to key enzymes on ascorbic acid stability as the ascorbate oxidases. However, the ascorbic acid reduction after US processing (e.g., 40 min; 42 kHz; <34 °C) were still far lower (≈2-fold lower) than those of conventional heat treatments (80 °C for 10 min) [91].

### 5.5. Cold Plasma

Cold plasma processing is also able to induce the enhancement of several phytochemicals. Cold plasma consists of an ionized gas (carbon dioxide, argon, nitrogen, helium, oxygen, or air) including active particles such as electrons, ions, free radicals and atoms. These reactive species have sufficient electrical energy to disrupt the covalent bonds of phytochemical compounds and cell membranes, which promotes their release, leading to higher contents of free phytochemicals. The reactive species generated, and therefore their effect on the phytochemical contents, depends on the parameters of the plasma source, such as its voltage, frequency, and plasma generation system, as well as the type of gas and its flow rate.

In particular, the optimal conditions to enhance the TPC of sour cherry juice with atmospheric plasma jet (single-electrode) at 2.5 kV were 3 min, 2.8 mL of sample volume and 0.75 L/h gas (argon) flow, reaching a TPC enhancement of 15% [103]. In the same study, increasing sample volume (3.2 mL) and gas flow (1.25 L/h) while maintaining treatment time (3 min) reached a total anthocyanin enhancement in sour cherry juice of 34% [103]. The authors attributed the higher anthocyanin increases to the presence of undefined small agglomerates or particles in the juice that could be dissociated during plasma processing. Furthermore, the anthocyanin stability might have been increased due to intra- and intermolecular association with other anthocyanins, or through pigmentation with copigments like flavonoids and hydroxycinnamic acids. In addition, the previous authors found that the TPC of pomegranate juice was increased up to ≈50% after plasma jet (single electrode) for 5 min, at 1 L/min argon for 3 mL of the sample [102]. Hydroxycinnamic acids seem to have higher stability than anthocyanins, as observed in chokeberry juice after single-electrode plasma jet processing [97]. The authors reported that hydroxycinnamic acids react less with radical species generated by the plasma, since they are less effective in reducing reagents, leading to their observed higher stability. Higher voltage (11 kV) during plasma jet (single electrode) using oxygen (1%)-argon at 1 L/min with longer treatment time (6 min) led to maximum TPC enhancements (7% higher) in blueberry juice [96]. Dielectric barrier discharge plasma (60 kV) of 100 mL sample (coconut–vegetable juice) for 10 min induced an enhancement of the TPC of only ≈5% [92]. With respect to the plasma orientation of the sample, atmospheric direct plasma (dielectric barrier discharge) application was more effective than indirect application in preserving the TPC, and consequently the TAC, than indirect application [101].

Increasing gas flow led to higher phenolic retention, leading to 14–28% higher TPC in cashew apple juice, with the maximum levels for 50 mL/min and longest treatment (15 min) [94]. Nevertheless, the authors agreed that flavonoids might require less energy to be released from their bonds than polyphenols, since lower flavonoid retention was achieved with the same plasma treatments regardless of gas flow or treatment time [94].

The highest increases of phenolic content with cold plasma were reported using spark discharge (10.5 kV) for 4–5 min with increases of 64–69% of the TPC of cloudy apple juice [95]. Such high phenolic enhancement may also be explained by the high PPO inactivation (70–80%) achieved with that cold plasma treatment [95]. The authors also stated, in agreement with previous literature, that spark discharge induces a higher concentration of some radical species (H_2_O_2_ and NO_3_^−^), which may induce higher enzyme inactivation [95]. Furthermore, such high phenolic enhancement could be attributed to this cold plasma method, which might increase cell membrane breakdown compared with other methods. Nevertheless, further work is needed to study phenolic changes in beverages using different cold plasma methods.

Although vitamin C is among the most labile phytochemicals during beverage processing, cold plasma has little effect compared with conventional heat treatments. Hence, short (2 min) cold plasma treatment (single-electrode plasma jet) with different oxygen concentrations (up to 1%) showed higher vitamin C retention than heat treatment (85 °C, 15 min) in blueberry juice [96]. The authors also found higher vitamin C retention even after longer cold plasma treatments (up to 6 min), when the oxygen concentration of the ionized gas was 0% [96]. Hence, prevention of vitamin C oxidation during cold plasma treatment was also studied in orange juice, with better vitamin C retention using air atmosphere compared with an oxygen-rich atmosphere (65% O_2_, 30% N_2_, 5% CO_2_) [100]. As observed, oxidation reactions initiated by reactive oxygen (and nitrogen) species are the most important reactions responsible for the high microbicidal effect of cold plasma processing, although it may have a negative effect on vitamin C stability [93,96,98,99,100]. Hence, apple juice treated with cold plasma (indirect plasma field; 80 kHz) under nitrogen gas flow led to better vitamin C retention, with levels increased by up to 11% [94]. The authors explained this vitamin C increase after cold plasma processing as being due to dehydroascorbate reductase (the enzyme responsible for the dehydroascorbic acid conversion back to ascorbic acid) activation triggered by some RNS (mainly nitric oxide), which are usually generated during cold plasma treatment [94].

### 5.6. Combined Technologies

The combination of processing technologies to obtain additive or synergistic effects is an effective strategy to improve their individual effects on product quality [12,57,115]. Hence, high microbial inactivation (up to 5.8 log reduction of *S. cerevisiae*) of individual US and PEF were enhanced when a combined treatment (30 min US + 60 s PEF) was used in apple juice [106]. Similarly, combined US (120 W for 5 min) + UV-C (20.2 kJ/m^2^) treatment achieved up to 5 log reductions of *Alicyclobacillus acidoterrestris* in apple juice [107].

Moreover, the application of combined technologies is an excellent strategy for enhancing the phytochemical content of beverages. Due to the high effectiveness of Ultrasound to preserve food quality and increase the extractability of phytochemicals, and to its relatively easy application in beverages, most studies on combined technologies for the treatment of beverages include US. For example, cold plasma (dielectric barrier discharge; 70 kV, 3 × 4 min) combined with US (750 W, 3 min) increased the individual effects of these technologies up to 22% and 34% for TPC and total carotenoid content, respectively, in carrot juice [109]. In particular, the lycopene and lutein contents of carrot juice were enhanced approximately four-fold and two-fold with combined cold plasma + US treatment [109].

US (18 kHz, 5 min) was applied in combination with HPP (450 MPa, 5 min) in prebiotic cranberry juice, with an increase of anthocyanins ranging from 14% to 20% being observed when the power level of 1200 W/L was used; meanwhile 600 W/L did not show the same benefits [110]. In addition, the same US-HPP treatment increased the contents of some fructooligosaccharides (DP4-nystose and DP5-1-fructofuranosylnystose), regardless of the power level (600 or 1200 W/L) [110]. A combination of HPP (300 MPa) and UV (28.7 J/mL) also reduced the pectinmethylesterase activity of cloudy apple juice from 190% relative activity (after increase due to single HPP treatment) to 60% [108]. A combination of US (20 kHz, 10 min) with UV increased the ascorbic acid, TPC and total carotenoid content of mango juice by approximately 47%, 35% and 200%, respectively [111]. Furthermore, mango juice processed with this combined treatment achieved better retention of these phytochemicals during cold storage, together with a higher bioaccessibility of the compounds due to the higher availability of these compounds in the treated mango juice [111].

As observed, the combination of these non-thermal technologies highly preserved and even increased the analysed content of several phytochemicals in fruit and vegetable beverages. This may be explained by the higher extractability of phytochemicals by these technologies, in particular US and HPP due to phenomena like cavitation, cell disruption, etc. In addition, the reduction of dissolved oxygen in the beverages due to cavitation during US treatment allowed for high preservation of these compounds from oxidation (of high interest for ascorbic acid). Thus, beverages treated with combined technologies may have better quality (e.g., lower colour differences due to enzymatic inactivation) [112] and higher contents of available phytochemicals (see Table 1), while ensuring food safety requirements.

## 6. Fortification of Beverages Using Natural Products

Fortification is a technique used to directly enrich the nutritional, bioactive and health-promoting compounds of beverages. However, the stability of the added components may be altered (oxidations, reactions with other components, etc.) due to the composition (organic acids, etc.) and physicochemical properties (pH, dissolved oxygen, etc.) of the beverage. Table 2 summarizes the fortification strategies of beverages by using natural products, which are also commented on in this section as follows.

### 6.1. Fortification of Beverages by Adding Plant Extracts and Other Health-Promoting Compounds

As is known, plants are a rich source of phytochemicals with great health-promoting properties. Such compounds may also be used for technological purposes, such as antimicrobials (extending microbiological shelf life and ensuring food safety), antioxidants (e.g., less quality degradation due to enzymatic systems), masking of undesired flavours (e.g., sucrose masks the bitter flavour of olive oil added into beverages), etc. In addition, the phytochemical content may be higher in the inedible plant parts (known in the food industry as by-products) compared with the edible parts. Therefore, their reuse becomes very important for the circular economy in the food industry, while minimising environmental impact and revalorization of phytochemicals for further use.

Fortification with added plant extracts leads to an almost proportional (to the added extract/compound quantity) increase in the level of beverage phytochemicals. However, interactions between different compounds (e.g., phenolic compounds) may lead to synergistic effects, as previously stated [124]. Nevertheless, special attention must be paid when adding some plant extracts (either by their sensory characteristics or the high concentrations used) since the sensory acceptance of the fortified beverage may be compromised [135,138].

In general, all studies published about beverage fortification by the addition of plant extracts have observed increases of their antioxidant properties, in most of cases related to their high phenolic compound contents (Table 2). Thus, beet leaf extract added to a fruit–vegetable smoothie (30% of total smoothie volume) increased both TPC and TAC by 50% [130]. Pale or black brewers’ spent grain extract added (10%) to cranberry juice increased TAC by 120% [124]. Similarly, pomegranate peel dry extract (2.5 mg per mL of beverage) and clove extract (200 μg per mL of beverage) added to carrot juice and cucumber juice, respectively, enhanced the TPC, TAC and flavonoid contents of the beverages [120,133]. Fortification of the carotenoid (lycopene and β-carotene) level of tomato juice was achieved by the addition of a polyphenol extract (0.5% of the beverage) from tomatoes. Finally, fortification of beverages with vitamins from plant extracts prior to processing is an excellent strategy to counterbalance subsequent vitamin degradation (of high interest for very labile vitamins) during processing or subsequent storage of beverages. Thus, the vitamin C and vitamin A contents of a fruit smoothie were increased by the addition (8% of the beverage) of mint leaf extract [140].

Another strategy for beverage fortification is pre-enhancement of the phytochemical content in the plant raw materials used for the subsequent obtaining of the beverage. For this, several green (chemical-free) postharvest abiotic stresses have been used, such as wounding, UV-C, and hyperoxic atmospheres (Table 2). Thus, a carrot smoothie prepared from pre-incubated carrots under hyperoxia treatment (80 kPa O_2_) increased the TPC of the smoothie by 2060% [121]. Similarly, the chlorogenic acid content of carrot juice was enhanced by 3600% when the unpeeled carrots were previously blanched (80 °C for 6 min) [122].

Fortification of beverages with pure compounds extracted from natural sources (plants and fish) have been also studied. The addition of some compounds may better preserve the phytochemicals contained in beverages. For example, the anthocyanin content of a blackberry juice was better retained for five weeks at 30 °C when glutathione was added (500 mg/L) [118]. Fortification of melon juice with (−)epicatechin (2.5 g/mL of the beverage) increased the TPC by ≈720% [128].

In this sense, Tarazona-Díaz & Aguayo [141] studied the effects of acidification, pasteurization, centrifugation, and refrigerated storage of watermelon juice (Table 2). Their results showed a minimal degradation of non-centrifuged juices stored at 4 °C, with particular richness in lycopene, polyphenols, and citrulline [141]. Indeed, the enrichment of Fashion watermelon juice by addition of L-citrulline (3.45 g per 500 mL) has shown to diminish muscle soreness perception from 24 to 72 h after a half-marathon race and to maintain lower concentrations of plasma lactate after exhausting exercise in amateur male runners [142]. Furthermore, lower (1.17 g per 500 mL) and higher (4.83 g per 500 mL) doses of L-citrulline in watermelon juices have reduced recovery heart rate and muscle soreness after 24 h muscle relief in athletes [143], which seems to be enhanced in combination with 22 mg pomegranate ellagitannins per 200 mL watermelon juice [144].

Nevertheless, special attention must be paid to the supplemented concentration since phytochemicals have limited bioavailability in the human body. For example, absorption of polyphenols may be relatively poor, ranging widely from 0.3 to 43%, leading to low circulating plasma concentrations [145]. For this reason, folic acid has been encapsulated with mesoporous silica particles (to be incorporated in fruit juices) in order to improve its stability, reduce the quantity of compound needed, and control release after consumption by modifying vitamin bioaccessibility [116]. Likewise, 16% of eicosanoid acid and 11% of docosahexaenoic acid were released in pomegranate juice when 0.1% of fish oil, microencapsulated by complex coacervation, was added to the juice [136].

### 6.2. Fortification of Beverages by Adding Algae

The culinary and health-promoting properties of marine algae, or seaweeds, have been known by Oriental cultures (mainly in Japan, China, and Korea) for centuries. In Western countries, algae use in pharmaceuticals, cosmetics, and food (mostly as thickening agents), together with its increasing acceptance as a culinary condiment, is growing day by day. Marine algae are rich sources of proteins, polysaccharides, minerals, vitamins and polyphenols, among other things [146,147], so their inclusion in beverage formulations may increase the phytochemical levels of those beverages [127]. More interestingly, these beverages may be fortified with unique health-promoting compounds of algae which are not found in plant products, for example, phlorotannins and fucoidans, among others [147]. However, special attention must be paid to the algae quantity added into the fortified beverages, since undesirable algae-related nuances may be detected. Thus, among nine green smoothies prepared with different marine algae, kombu- and wakame-fortified beverages showed the lowest sensory scores, mainly due to off-odours related to those algae [127].

Fortification of a fruit-beverage smoothie with microalga *Dunaliella salina* (2.5% of the beverage) increased the TPC and TAC while still being scored with great sensory quality [137]. Furthermore, the total sugar content of date nectar fortified with spirulina (10%) was increased, which is of high interest in masking some undesirable flavours, such as the bitter flavour of kale beverages [148]. The authors observed better sensory attributes in the date nectar fortified with spirulina.

### 6.3. Phytochemical Fortification of Beverages during Fermentation

Probiotics are living organisms whose ingestion provides several health benefits, including prevention of gastrointestinal diseases, promotion of antimicrobial activity, regulation of lactose metabolism, decrease in serum cholesterol levels, stimulation of the immune system, and anti-mutagenic and anti-carcinogenic effects, among others [131]. Lactic acid bacteria are widely used during food processing for fermentation, which gives typical flavours and other sensory aspects. Hence, much use of probiotics (e.g., *Lactobacillus, Bifidobacteria*, etc.), for fermentation and/or fortification purposes is made in the food industry to provide the consumer with beverages that have excellent quality and enhanced health-promoting properties.

The use of probiotics in beverages may also enhance their phytochemical content. For example, the phenolic and anthocyanin levels of blueberry juice were increased by 43% and 15%, respectively, after fermentation (24 h at 37 °C followed by 2 h at 4 °C) with *Lactobacillus plantarum* [119]. TPC and TAC were increased (by 6–8-fold) in different fruit juices after fermentation (2 h at 30 °C followed by 28 days at 4 °C) with *L. plantarum* [126]. Similarly, TPC as well as flavonoid and anthocyanin contents were increased in fruit–vegetable juice after fermentation (24 h at 37 °C followed by refrigeration at 4 °C) with *L. plantarum* [131]. Other *Lactobacillus* species also induced increases of phenolic (49% TPC increase after fermentation with *Lactobacillus paracasei*) [123], TAC (74% increase after fermentation with *Lactobacillus acidophilus*) [134] and other compounds like riboflavin, β-carotene and sulforaphane (broccoli juice fermented with *Pedioccoccus pentosaceus*) [132]. Probiotic survival under gastrointestinal conditions may be limited, with the strains being used having higher resistance to those conditions (acidic pH, enzymatic reactions, etc.); microencapsulation of probiotics to extend their survival may also be effective [117,131].

As observed, fermentation of beverages with probiotic bacteria may lead to large increases in their phytochemical contents, mainly of phenolic compounds. Such enhancement has been hypothesized to occur due to the disintegration of macromolecular polyphenol or anthocyanin structures into smaller phenols; this also increases their bioaccessibility through the specific metabolism (e.g., deglycosylation) of probiotic bacteria [119,131].

## 7. Conclusions

Fortification of fruit and vegetable beverages can be achieved by using green non-thermal technologies. While ultrasound is already a well-known technology used for cell disruption, UV and high-pressure processing may lead to similar increases in such beverage phytochemicals as phenolic compounds, anthocyanins, vitamin C, etc., ensuring high antioxidant properties. Pulsed electric fields and cold plasma are also green non-thermal technologies that are used to extend the shelf life of fruit and vegetable beverages; however, they are also capable of being used to fortify the phytochemical contents of these food products. Our analysis of published studies on this topic shows that these are excellent strategies to fortify the health-promoting compound contents of the plant products contained in these beverages. In addition, the optimized combination of the technologies used for treatment may highly enhance fortification rates, as most of the available literature relates to the combination of such new techniques with ultrasound. Based on this assessment, future studies may deepen understanding of different combinations of such technologies where optimal processing conditions may differ depending on beverage properties (rheology, composition, pH, etc.). Furthermore, a deeper elucidation should be sought as to whether the fortification observed with these technologies is due more to higher extractability after cell disruption or to biosynthetic reactions, for example, when substrate and enzymes come in contact.

## Figures and Tables

**Table 1:** Effects of green processing technologies on the phytochemical profiles of juice and smoothie beverages, along with other quality attributes.

Technology	Beverage Type and Components	Treatment Conditions	Optimum Conditions	Shelf Life	Main Results Obtained	Reference
ULTRAVIOLET	Black carrot juice	254 and 365 nm; 15 W LMP lamps: 2.16·10^−3^ and 1.50·10^−3^ kJ/m^2^ respectively; 0–60 min; 25 °C	365 nm 1.5·10^−3^ kJ/m^2^	-	Increased TPC No effect on colour Approx. 1 log reduction in microbial spoilage	[65]
	Carrot juice	30 W LMP lamps; 11.4 kJ/m^2^	253.7 nm 11.4 kJ/m^2^	12 d at 5 °C	Good sensory parameters during storage	[67]
	Carrot-carob-ginger-lemon-grape juice	280 and 365 nm; 0.6–0.4 mW LEDs; single and combined wavelength for 10–100 min; 25 °C	Combined 280/365 nm 0.77/2.2 kJ/m^2^	-	Increased TPC and TAC 4.05 log reductions of inoculated *Escherichia coli* Minimum effect on physical properties	[66]
	Carrot-orange juice	30 W LMP lamps; 0–10.6 kJ/m^2^; 0–15 min; 1.6 L/min; 20 °C	253.7 nm 10.6 kJ/m^2^	-	Up to 2.5–5.9 log reductions in inoculated *E. coli*, *Pseudomonas fluorescens*, and *saccharomyces cerevisiae*	[62]
	Kale juice	25 W LMP lamps: 74 and 108.3 mJ/cm^2^; 0.14 L/min; RT	253.7 nm 1.08 kJ/m^2^	4 d at 4 °C	Reduction by 20% in TPC Up to 5-log reduction of inoculated *E. coli* No effect on viscosity, chlorophyll content, colour, TAC, PPO, and POD Increased sedimentation rate Higher PME activity	[61]
	Melon juice	15 W LMP lamps; 4 and 16 kJ/m^2^; 5 and 20 min; 25 °C	254 nm 16 kJ/m^2^	13 d at 5 °C	Better retention of TAC and colour No effect on TPC Moulds and yeasts did not grow	[63]
	Pineapple-mango juice	55 W LPM lamps; 0.08 kJ/m^2^; 8.65 s	254 nm; 8 kJ/m^2^	9 weeks at 4 °C	Minimal degradation of ascorbic acid TPC and TAC retention	[64]
HPP	Apple-carrot-zucchini-pumpkin-leek smoothie	350 MPa; 10 °C; 5 min	350 MPa 10 °C 5 min	28 d at 4 °C	High retention of vitamin C during storage Retention of antioxidants (TPC and flavonoids) Lower microbiological load Higher oxidation due to earlier clarification	[68,69]
	Apple-orange-strawberry-banana smoothie	350–600 MPa; 10 °C; 3–5 min	350 MPa 10 °C 5 min	48 h at 4 °C	Does not affect phenolics and flavonoids Preserves vitamin C and flavours Ensures microbial quality	[70]
	Apple-strawberry-banana-orange smoothie	450–600 MPa; 20 °C; 5–10 min	600 MPa 20 °C 5 min	10 h at 4 °C	Retains ascorbic acid High PPO inactivation rate (83%)	[71]
	Berries-grape-orange-strawberry-apple smoothie	100–300 MPa; −5–45 °C; 5 min	300 MPa 45 °C 5 min	15 d at 4 and 20 °C	Reduction of up to 6-log of mesophilic lactobacilli	[72]
	Carrot juice	550 MPa; <38 °C; 6 min	550 MPa <38 °C 6 min	20 d at 4 °C	Better carotenoid (α- and β-carotene), phenolic, polyacetylene and TAC retention Better preservation of nutritional compounds Higher rheological properties Better sensory attributes	[73]
	Carrot-pumpkin smoothie	300–600 MPa; 23 °C; 5 min	400 MPa 23 °C 5 min	7 d at 5 °C	Mild TPC reduction (<15%) Better TPC preservation during storage High microbial control (≈6 log lower counts) No high physicochemical changes (SSC, pH and colour)	[74]
	Grape juice	500 MPa; 45 °C; 5 min	500 MPa 45 °C 5 min	-	Reduction of 17–29% in aflatoxins	[75]
	Indian gooseberry juice	200–500 MPa; 30–60 °C; 5 min	500 MPa 30 °C 5 min	-	Increase in TPC and TAC up to 50 °C Less vitamin C degradation	[76]
	Juçara-mango juice	600 MPa; 25 °C; 5 min	600 MPa 25 °C 5 min	-	Does not affect anthocyanin content Good sensory properties	[77]
	Orange juice	0–200 MPa; 25 °C; 1 min (whole peeled orange) + 400 MPa;40 °C; 1 min (juice)	200 MPa 25°C 1 min + 400 MPa 40 °C 1 min	-	Non-additive effect in flavonoids and vitamin C 12-fold increase in content of colourless carotenoids	[78]
	Tomato-pepper-celery-cucumber-onion-carrot-lemon beverage	100–400 MPa; <30 °C; 2–9 min	400 MPa <30 °C 2–5 min	-	Good preservation of vitamin C Slight colour change	[79]
PEF	Apple juice	20–30 kV/cm; 5–125 µs; ≤55 °C	25 kV/cm 63 µs ≤55 °C	-	>5 log reduction cycles of *E. coli*, *L. monocytogenes*, *Staphylococcus aureus*, and *Salmonella typhimurium*	[80]
	Apple-strawberry-banana smoothie	13.5–24 kV/cm; 100–290 Hz; 8.7–24.1 pulses; 3 µs; 130 L/h; <58 °C	24 kV/cm; 100 Hz 8.7 pulses <58 °C	27 d at 7 °C & 7 d at 4 °C	Highest inactivation of moulds and yeasts	[81]
	Grape juice	3 kV/cm; 238 pulses up to 500 kJ/kg; sample: 215 mL; <75 °C	3 kV/cm 238 pulses <75 °C	-	Reductions by 24–84% in aflatoxins	[75]
	Grapefruit juice	20 kV/cm; 1 kHz; 80 mL/min; <45 °C	20 kV/cm; 1 kHz <45 °C	-	Lower non-enzymatic browning and viscosity than the untreated sample	[82]
	Mango-papaya-stevia juice	20–40 kV/cm; 2.5 µs; 30 mL/min; 100–360 µs; <50 °C	21 kV/cm 360 µs <50 °C	-	Greatest content of bioactive compounds Minimal colour changes	[83]
ULTRASOUND	Apple juice	2 W/cm^2^; 25 kHz; 70%; 30–60 min; 20 °C; sample: 60 mL	2 W/cm^2^; 25 kHz 30 min 20 °C	-	The highest polyphenolic and sugars content Higher minerals and total carotenoids (60 min)	[84]
	Apple-carrot-stevia juice	750 W; 20 kHz; 20–80%; 15 min; sample: 100 mL	750 W; 20 kHz; 60% 15 min	-	Better phenolic profile Better radical scavenging activity	[85]
	Apple-strawberry-banana-orange smoothie	1.5 kW; 20 kHz; 40–100%; 25 °C; sample: 200 mL	1.5 kW; 20 kHz; 70% (42.7 μm) 3 min 25 °C	-	Better TPC preservation Higher flavonoid content	[86]
	Carrot juice	750 W; 20 kHz; pulses of 5 s on and 5 s off; 70%; 15 °C; sample: 250 mL	750 W; 20 kHz; 70% 15 °C	48 h at 4 °C	Enhancement of colouring pigments, sugar, chlorogenic acid and some mineral contents Decreased microbial population	[87]
	Grape-apple juice	750 W; 20 kHz; 100%; 20–40 min; sample: 100 mL	750 W; 20 kHz; 100% 20 min	-	Increased phenolic profile and TAC Higher organic acids	[88]
	Grapefruit juice	720 W; 28 kHz; 70%; 30–90 min; 20 °C	720 W; 28 kHz; 70% 90 min 20 °C	-	Improvement in sugar, carotenoid, mineral and phenolic content Decreased spoilage microbe population	[89]
	Strawberry-banana-juçara smoothie	73.5–250 W; 20 kHz; 7–19 min; <60 °C; sample: 200 mL	147 W; 20 kHz 2 min <60 °C	-	The highest anthocyanin retention	[30]
	Orange juice	33.31 W/mL; 24 kHz; 105 µm; 1–30 min; <46 °C; sample: 30 mL	33.31 W/mL; 24 kHz; 105 µm 30 min <46 °C	28 d at 5 °C	Increased phenolic and flavonoid content Higher vitamin C retention Better sensorial properties	[90]
	Nopal beverage	240 W; 42 kHz; 10–40 min; <34 °C; sample: 300 mL	240 W; 42 kHz 40 min <34 °C	28 d at 4 °C	Higher stability for bioactive compounds Ascorbic acid reduction Best acceptability Low changes in colour	[91]
	Tomato-coconut water-beetroot juice-based beverage	240 V; 37 kHz; 10 and 15 min	240 V; 37 kHz 10 min	-	High sinapic and gallic acid contents Ascorbic acid reduction 1 log reduction of yeast and mould	[92]
COLD PLASMA	Apple juice	Atmospheric jet; 65 V; 1.1 MHz; 0–0.1% oxygen-argon gas flow: 5 slm; 0–8 min	Atmospheric jet; 65 V 0.1% O_2_ in Ar gas 8 min	24 h	Reduction of *C. freundii* by ~5 log cycles	[93]
	Apple juice (cashew)	Indirect plasma field under 30 kPa; 80 kHz; nitrogen gas flow: 10–50 mL/min; 5–15 min; sample: 10 mL	Indirect plasma; 30 kPa 10 mL N_2_/min 5 min	-	Increased TPC and TAC Higher vitamin C retention	[94]
	Apple juice (cloudy)	Spark and glow discharge; 7.9–10.9 kV; 20–65 kHz; 1–5 min	Spark discharge 10.5 kV 5 min	28 d at 4 °C	Increased TPC and TAC PPO inactivation Lighter juice colour	[95]
	Blueberry juice	Single-electrode atmospheric jet; 11 kV; 1 kHz; 0–1% oxygen-argon gas flow: 1 L/min; 2–6 min	Single-electrode 11 kV 1% O_2_ in Ar gas 6 min	-	Increased TPC and TAC Higher content of anthocyanin and vitamin C (at 0% O_2_ and 2–4 min) than heat treatment 7.2 log reduction of Bacillus	[96]
	Chokeberry juice	Single-electrode atmospheric jet; 25 kHz; argon gas flow: 0.75 dm^3^/min; time: 3–5 min; 24 °C	Single-electrode 5–7 cm^3^ 3 min	-	Polyphenolic content stability	[97]
	Coconut liquid endosperm	Atmospheric jet powered by a microwave generator; 450–650 W; air gas flow: 5 L/min; time: 0–25 min	Atmospheric jet 450 W 22–24 min	-	Reduction of initial counts of *S. enterica* and *E. coli* by 4 log cycles	[98]
	Orange juice	DBD-low-temperature plasma; 30 kV; 60 kHz; time: 3–12 s and 5–20 s; sample: 50 µL and 4 mL, respectively	DBD; 30 kV 10 s	16 d at 4 °C	Absence of *E. coli* in juice inoculated with 4.20 × 10^7^ CFU/mL Preservation of vitamin C content	[99]
	Orange juice	DBD-atmosphere CP; 90 kV; 60 Hz; time: 30–120 s; sample: 25–50 mL; atmosphere gas: air or 65% O_2_	DBD; 90 kV 2 min direct plasma 50 mL 65% O_2_	24 h at 4 °C	Reduction of 4.7-log of *S. enterica* Reduced PME enzyme activity Higher vitamin C retention in air-packaging compared to the high-oxygen atmosphere.	[100]
	Orange juice with oligosaccharides	DBD-atmosphere CP (direct and indirect plasma field); 70 kV; 50 Hz; time: 15–60 s; sample: 20 mL	DBD 70 kV Direct plasma field	24 h at RT	12% oligosaccharide loss preservation of TPC, TAC and colour	[101]
	Pomegranate juice	Single-electrode atmospheric jet; 2.5 kV; 25 kHz; argon gas flow: 0.75–1.25 dm^3^/min; time: 3–5 min; sample: 3–5 cm^3^	Single-electrode; 2.5 kV 0.75 dm^3^/min 3 min 5 cm^3^	-	Greater anthocyanin stability Less colour changing with higher gas flow	[97]
	Pomegranate juice	Single-electrode atmospheric jet; 2.5 kV; 25 kHz; argon gas flow: 0.75–1.25 dm^3^/min; time: 3–5 min; sample: 3–5 cm^3^	Single-electrode; 2.5 kV 1 dm^3^/min 5 min 3 cm^3^	-	Better phenolic compound stability	[102]
COLD PLASMA	Sour cherry Marasca juice	Single-electrode atmospheric plasma jet; 2.5 kV; 25 kHz; argon gas flow: 0.75–1.25 L/min; time: 3–5 min; sample: 2–4 mL	Single-electrode; 2.5 kV 3 min 3 mL	-	Highest anthocyanin and phenol content	[103]
	Tomato juice	DBD; 10 kV; 5 min; 30 °C	DBD; 10 kV 5 min 30 °C	-	No effects on flavour and aroma Lower volatile compound release	[104]
	Tomato-coconut water-beetroot juice-based beverage	DBD; 60 kV; 50 Hz; time: 10 and 15 min; sample: 100 mL	DBD; 60 kV 10 min	-	Improvement of TPC	[92]
	White grape juice	DBD; 80 kV; 60 Hz; time: 1–4 min; 24 °C	DBD; 80 kV 1 min 24 °C	-	Higher bioactive compound levels	[105]
COMBINED TECHNOLOGIES	Apple juice	US + PEF US: 600 W; 20 kHz; 80%; 20–44 °C; 10–30 min; sample: 95 mL PEF: 23.9–71.6 J/cm^2^; 360 µs; 2–60 s; sample: 5 mL; <56 °C	US30 + PEF60	15 d at 5 °C	5.8-log reduction of *S. cerevisiae*	[106]
	Apple juice	UV + US; US + UV UV: 254 nm; 15 W lamps: 13.44 W/m^2^; 5–25 min US: 120–480 W; 35 kHz; 5–25 min	US: 120 W; 5 min + UV: 254 nm; 20.2 kJ/m^2^	-	5-log reduction of *A. acidoterrestris*	[107]
	Apple juice (cloudy)	HPP + UV HPP: 0–300 MPa; 32 °C; sample: 13 L UV: 254 nm; 55 W lamp; 14.3–28.7 J/mL; 20 °C; sample: 70 mL	HPP: 300 MPa; 32 °C + UV: 254 nm; 28.7 J/mL	-	Increased TPC by 277.6% Reduced PME activity	[108]
	Carrot juice	CP + US CP: DBD; 70 kV; time: 3 × 4 min, US: 750 W; 20 kHz; pulses of 5 s on and 5 s off; 80%; <20 °C; 3 min; sample: 100 mL	CP: DBD; 70 kV + US: 750 W; 3 min; <20 °C	-	Better stability Higher TPC, carotenoid, lycopene, and lutein Up to 2 log reductions of mesophilic and yeast and moulds	[109]
	Cranberry juice	US + HPP US: 600–1200 W/L; 18 kHz; <25 °C; 5 min HPP: 450 MPa; 11.5 °C; 5 min	US: 1.2 kW/L; 5 min; 25 °C + HPP: 450 MPa; 5 min; 11.5 °C	-	Higher anthocyanin content Good preservation of FOS	[110]
	Mango juice	US-UV: 600 W; 20 kHz; pulses of 5 s on and 5 s off; 10 min; 3600 J/mL; sample: 100 mL UV: 254 nm; 8 W lamp;	US-UV: 600 W; 254 nm; 3.6 kJ/mL; 10 min	30 d at 4 °C	Increased the bioaccessibility of ascorbic acid, TPC, and carotenoids by 102%, 114%, and 32%, respectively Good retention up to 30 d	[111]
	Orange juice	US + PEF US: 500 W; 30 kHz; 55 °C; 10 min; sample: 800 mL PEF: 40 kV/cm; 15 Hz; 100 µs	US: 500 W; 10 min; 55 °C + PEF: 40 kV/cm	168 d at 25 °C	Lower colour differences Similar attributes to heat treatment	[112]

TPC: total phenolic content; TAC: total antioxidant capacity; PPO: polyphenol oxidase; POD: peroxidase; DBD: dielectric barrier discharge; RT: room temperature; FOS: fructooligosaccharides; PME: Pectin methylesterase.

**Table 2:** A review of the main phytochemical fortification conditions in beverages.

Beverage Type and Components	Fortification Conditions	Optimum Conditions	Shelf Life	Results	Reference
Apple and orange juices	Encapsulation of folic acid (synthesised) in mesoporous silica particles	Encapsulated	-	Improved stability Controlled release after consumption by modifying vitamin bioaccessibility	[116]
Apple and orange juices	Free and microencapsulated *L. acidophilus* (10 and 30%)	Microencapsulated	63 d at 4 °C	Extended survival	[117]
Blackberry juice	Glutathione, galacturonic acid, diethylenetriaminepentaacetic acid, and tannic acid (500 mg/L)	Glutathione	5 weeks at 30 °C	Great retention of the anthocyanin content	[118]
Blueberry juice	*Lactobacillus plantarum* strain J26	*L. plantarum*	-	Increased TPC by 43% Increased anthocyanin content by 15%	[119]
Carrot juice	Pomegranate peel extract obtained by HPP (0–2.5 mg/mL)	2.5 mg/mL	42 d at 4 °C	Higher TAC Improved microbial safety	[120]
Carrot juice	Carrot shreds under combination of UV-C (4 kJ/m^2^) and 72 h at 15 °C in air or hyperoxia (80 kPa O_2_) conditions	Non-UV-C + 80 kPa O_2_ (72 h at 15 °C)	14 d at 5 °C	Increased TPC by 2060% Good microbial quality	[121]
Carrot juice	Carrot slices, peeled and unpeeled (48 h at 15 °C) + blanching (80 °C; 6 min)	Unpeeled carrot slices + blanching	-	Increased by 3600 and 195% in chlorogenic and TPC, respectively Increased minerals by 7–40%	[122]
Chokeberry juice	*Lacticaseibacillus paracasei* strain SP5 (10 g/mL)	*L. paracasei*	4 weeks at 4 °C	Increased TPC by 49%	[123]
Cranberry juice	0–10% (*v/v*) of pale brewers’ spent grains (BSG) 0–10% (*v/v*) of black BSG	10% of pale or black BSG	-	Increased TAC by 120%	[124]
Date-puree nectar	Spirulina (0–20%)	10% of spirulina	-	Higher total sugar and carotenoid contents Better sensory attributes	[125]
Emmer-based beverage	*Lactiplantibacillus plantarum* 2035 + blueberry, aronia or grape juice	*L. plantarum* + aronia juice	4 weeks at 4 °C	Higher TPC and TAC High viable counts during storage	[126]
Grape-broccoli-cucumber smoothie	2.2% of alga (sea lettuce, kombu, wakame, thongweed, dulse, Irish moss, nori, chlorella, or spirulina)	Chlorella and spirulina	24 d at 5 °C	The highest vitamin C content High fucose content for thongweed, kombu, and wakame-based smoothies	[127]
Melon juice	(−) Epicatequin (1.25–5 g/mL)	2.5 g/mL	10 d at 4 °C	Increased TPC by 724%	[128]
Multi-fruit juice byproducts	Ginger and apple (50:50, *w/w*) and apple, carrot, beet, and ginger (50:29:20:1, *w/w*) juice obtained by fresh and freeze-dried byproducts	Ginger and apple juice obtained by freeze-dried by-products	-	Higher TPC, TAC, and flavonoids	[129]
Orange- apple-carrot-beet smoothie	Beet leaf extract (30% *v/v*)	Beet leaf extract (30%)	21 d at 5 °C	Increased TPC content by 50% High TAC	[130]
Orange-celery-carrot-lemon juice	Fermentation by *L. plantarum* strain HFC8	*L. plantarum*	-	Low mesophilic aerobic bacteria and yeast and mould counts Increased TPC, flavonoid, and anthocyanin content	[131]
Pasteurised broccoli juice	Fermentation by *Pediococcus pentosaceus* (isolated from fermented cherry juice and pickled pig’s ear)	*P. pentosaceus* of animal origin	-	Increase riboflavin and β-carotene content Decrease free amino acids Both obtained high sulforaphane content	[132]
Pasteurised cucumber juice	Cinnamon, clove, mint, and ginger extracts (200–800 µg/mL)	200 µg/mL of clove extract	6 months RT	Better sensory attributes Higher TPC and flavonoid content Good retention for 6 months	[133]
Pasteurised peach juice	*Lactobacillus acidophilus* PTCC 1643 and *Lactobacillus fermentum* PTCC 1744	*L. acidophilus*	-	Inhibited Maillard reaction by 36.7%; 38.5% of anti-inflammatory activity Increased TAC by 74%	[134]
Pineapple- banana-apple smoothie	Moringa leaves (0–4.5%)	4.5% of moringa leaves	-	Increased vitamin C and E by 227% and 102%, respectively Highest TPC and TAC Lower sensorial quality	[135]
Pomegranate juice	0–0.1% of fish oil microcapsules by complex coacervation	0.1% fish oil microcapsules	42 d at 4 °C	16% of eicosapentaenoic acid and 11% of docosahexaenoic acid were released after 42 d Good sensory quality up to 0.07% microcapsules	[136]
Spinach-green apple-cucumber smoothie	2.5% of alga (*Chlorella vulgaris* and *Dunaliella salina*)	*D. salina*	28 d at 5 °C	Higher TPC and TAC Great sensory attributes	[137]
Strawberry- banana smoothie	Olive leaf extract (-OLE- 0–25 mg/100 g) + Sucrose (0–4 g/100 g)/sodium cyclamate (0–114.4 mg/100 g)/sodium chloride (0–40 mg/100 g)	OLE (20 mg/100 g) + sodium chloride (40 mg/100 g)	-	High TPC 40% less bitter taste perception	[138]
Tomato juice	Polyphenols from 0.5% of tomato extracts	Tomato extract (0.5%)	-	High TPC, lycopene, and β-carotene contents	[139]
Watermelon- apple-banana smoothie	Mint leaf extract (0–8%)	Mint leaf extract (8%)	-	High vitamin A, C, flavonoid, and TPC	[140]
Watermelon juice	Citric acid, malic acid, or lemon juice (pH = 3.8)	Non-centrifuged and addition of citric acid or lemon juice (pH = 3.8)	20 d at 4° C	Retention of sensory and functional qualities	[141]

TPC: total phenolic content; TAC: total antioxidant capacity; RT: room temperature.

## Data Availability

Not applicable.
